# Inhibition of Annexin A2 gene transcription is a promising molecular target for hepatoma cell proliferation and metastasis

**DOI:** 10.3892/ol.2013.1663

**Published:** 2013-11-06

**Authors:** ZHIZHEN DONG, MIN YAO, HAIJIAN ZHANG, LI WANG, HUA HUANG, MEIJUAN YAN, WEI WU, DENGFU YAO

**Affiliations:** 1 Research Centre of Clinical Medicine, Affiliated Hospital of Nantong University, Nantong, Jiangsu 226001, P.R. China; 2 Medical School of Nantong University, Nantong, Jiangsu 226001, P.R. China; 3 Department of Pathology, Affiliated Hospital of Nantong University, Nantong, Jiangsu 226001, P.R. China

**Keywords:** hepatocellular carcinoma, Annexin A2, upregulation, small hairpin RNA, hepatitis B virus, metastasis

## Abstract

Hepatocyte Annexin A2 (ANXA2) expression is associated with the progression and metastasis of hepatocellular carcinoma (HCC). Circulating ANXA2 levels in HCC patients are significantly higher compared with that of patients with benign liver disease. ANXA2 levels have been found to correlate with hepatitis B virus infection, extrahepatic metastasis and portal vein thrombus. By contrast, ANXA2 levels do not correlate with tumour size and AFP levels. However, the underlying mechanisms of ANXA2 remain obscure. The results of the current study identified that abnormalities in hepatic ANXA2 expression were localised to the cell membrane and cytoplasm of HCC tissues and mainly in the cytoplasm of para-cancerous tissues. ANXA2 was overexpressed in MHCC97-H cells which have high metastatic potential. Following specific ANXA2-small hairpin RNA (shRNA) transfection *in vitro,* ANXA-2 was effectively inhibited and the S phase ratio of cells was 27.76%, compared with 36.14% in mock-treated cells. In addition, the invading cell ratio was reduced in the shRNA-treated group (52.16%) compared with the mock-treated group (86.14%). The growth and volume of xenograft tumours *in vivo* was significantly suppressed (P<0.05) in the shRNA group compared with that of the mock group, indicating that ANXA2 may be a novel and useful target for elucidating molecular mechanisms involving the proliferation and metastasis of HCC.

## Introduction

Hepatocellular carcinoma (HCC) is the 3^rd^ leading cause of cancer-related mortality (1–3), particularly in the inshore area of the Yangtze River, China (4–6). Despite previous therapeutic advances, HCC continues to be a significant cause of cancer-related morbidity and mortality, which generally carries a poor prognosis (7,8). Surgical therapy with liver transplantation or resection remains the mainstay of curative therapy for patients in the early stages of HCC (9,10). Multiple pathogenic factors, including infection with hepatitis B virus (HBV) and hepatitis C virus (HCV), are major etiological agents of chronic liver disease and HCC and the subsequent multistage pathogenesis of HCC has been extensively investigated in previous studies (3,11,12). In addition, advances in molecular genetics have identified a large number of activated or suppressed genes which may be significant for hepatocarcinogenesis and HCC metastasis (6,13). However, it is unclear how these factors cause the progression to HCC.

Accumulating clinical results have indicated that Annexin A2 (ANXA2) promotes tumour metastasis by inducing the conversion of plasminogen to plasmin (14), alteration of molecular tyrosine 23 phosphorylation (15,16), interaction with HAb18G/CD147 (17), activation of matrix metalloproteinases (MMPs) and degradation of extracellular matrix (ECM) components (18). ANXA2 has been revealed as a multifunctional protein *in vitro* and *in vivo.* ANXA2 is located on the cell surface and is involved in biological processes, including anti-inflammatory effects, exocytosis, immune responses and phospholipase A2 regulation and is important for cell malignant transformation and progression for tumour invasion and metastases (19,20). ANXA2 expression and pathological features in HCC patients have been previously investigated (21), however, the correlation and underlying molecular mechanisms between abnormal ANXA2 and HCC growth remain unclear. The objectives of the present study were to analyse ANXA2 expression and gene transcription in hepatoma cell lines and focus on the effects of silencing ANXA2 by small hairpin RNA (shRNA) on the proliferation and invasion ability of hepatoma cells with high metastatic potential *in vitro* and *in vivo*.

## Materials and methods

### Tissues

Cancerous, para-cancerous and non-cancerous tissues were obtained from 30 patients who underwent surgery for HCC at the Affiliated Hospital of Nantong University (Nantong, China). Tissues were immediately frozen in liquid nitrogen and kept at 80°C until required. Cases included 22 males and 8 females and of these cases, 18 exhibited tumour sizes of ≥5 cm and 21 exhibited AFP levels of ≥ 50 ng/ml. Pathological examination with H&E staining showed that cancerous tissues were highly differentiated HCC. Of the para-cancerous tissues, 22 exhibited cirrhosis, 12 exhibited chronic hepatitis and 24 exhibited atypical hyperplasia. Of the non-cancerous tissues, 21 exhibited cirrhosis, 12 exhibited chronic hepatitis and 15 exhibited atypical hyperplasia. One portion of the specimen was for total RNA extraction and ANXA2 preparation and the other was fixed with 10% formalin for immunohistochemistry. Prior written informed consent was obtained from all patients according to the World Medical Association Declaration of Helsinki and the study received ethics board approval from the Affiliated Hospital of Nantong University.

### Cell culture, shRNA and transfection

shRNA corresponding to nucleotides 94–113 downstream of the transcription start site of the ANXA2 gene was synthesised according to methods previously described (22). The shRNA was inserted into *Bam*HI- and *Hin*dIII-linearised pRNAT-U6.1/Neo shRNA expression vectors and fragments were conformed by sequencing and named as pRNAT-U6.1-shRNA or -negative, respectively. HepG2, SMMC-7721, SMMC-7402 and LO_2_ cell lines were obtained from Biomics Biotechnologies (Nantong) Co., Ltd. (Nantong, China) and MHCC97-H cell lines were obtained from the Liver Cancer Institute, Fudan University (Shanghai, China). Cells were grown in DMEM with 10% fetal bovine serum at 37°C and 5% CO_2_ and grown to 90–95% confluency. MHCC97-H cells were transfected with the pRNAT-U6.1-shRNA or -negative plasmids using PolyJet™ (SignaGen, Rockville, MD, USA). At 48 h, selection was performed with medium containing 400 μg/ml G418 for 14 days, followed by 200 μg/ml G418. Cell lines were named as the shRNA or negative groups and blank cells were named as the mock group.

### RNA isolation and quantitative PCR (qPCR)

RNA was isolated from 50 mg liver tissue using TRIzol reagent (Life Technologies Corporation, Carlsbad, CA, USA) according to the manufacturer’s instructions. RNA integrity was examined by 1% agarose gel electrophoresis and quantity and purity was based on absorbance (A) at A_260_ and the ratio at A_260/280_ (Smartspec™ plus spectrophotometer; Bio-Rad, Hercules, CA, USA). ANXA2-cDNA was synthesised from 1 μg RNA using the 1^st^ strand cDNA synthesis kit (Fermentas Canada Inc., Burlington, ON, Canada). qPCR was performed using the StepOne™ system (Applied Biosystems, Foster City, CA, USA) with a solution containing 25 μl 2X SYBR Premix ExTaq (Takara Bio, Inc., Shiga, Japan), 2 μl primer mix, 1 μl 50X ROX Reference Dye I, 4 μl cDNA and 18 μl deionised water to 50 μl. The following primers were used: i) ANXA2 forward, 5′-TGAGCGGGATGCTTTGAAC-3′ and reverse, 5′-ATCCTGTCTCTGTGCATTGCTG-3′; and ii) β-actin forward, 5′-ATTGCCGACAGGATGCAGA-3′ and reverse, 5′-GAGTACTTGCGCTCAGGAGGA-3′ were used as control. In addition, a no template control was included in each run. The optimised conditions were as follows: 1 cycle at 95°C for 2 min, 40 cycles of 95°C for 10 sec, 62°C for 1 min and 60°C for 15 sec and analysis was performed using the 2^−ΔΔCt^ method.

### Immunofluorescence assay

Cells were cultured for 24 h on cover slips in 24-well plates, fixed with 4% paraformaldehyde and then blocked with PBS containing 3% BSA. Samples were incubated with rabbit anti-human ANXA2 antibody (1:100; Santa Cruz Biotechnology, Inc., Santa Cruz, CA, USA) overnight. Following incubation with Cy3-labelled goat anti-rabbit IgG secondary antibody (1:500; Beyotime Institute of Biotechnology, Haimen, China), cells were stained with 4′,6-diamidino-2-phenylindole (DAPI) and sealed with 50% glycerin. Observations were performed under a microscope (IX71; Olympus Corp., Tokyo, Japan).

### Cell proliferation, cell cycle and transwell assay

Cell proliferation was evaluated using a cell counting kit-8 (CCK-8; Beyotime Institute of Biotechnology). Cells and blank controls were seeded in 96-well plates (2×10^3^ cells/well with 100 μl medium; n=5) and cultured for 24 h. Next, 10 μl CCK-8 solution was added to the culture medium for 2 h and the absorbance was recorded at A_450_ by a microplate reader (BioTek Instruments, Inc., Winooski, VT, USA). This was repeated 5 times at various time points. The cell cycle assay was performed using a cell cycle and apoptosis kit (Beyotime Institute of Biotechology). Cells were seeded at 1.0×10^6^ cells/well (6-well plate) and cultured for 24 h. Subsequently, cells were digested with trypsin and fixed for 24 h at 4°C in pre-cooled 70% ethanol. Cells were stained with propidium iodide and analysed by a flow cytometry (BD FACSCalibur; BD Biosciences, Franklin Lakes, NJ, USA) for cell cycle analyses.

Cells were plated at 1.0×10^5^ cells/well in 0.5 ml serum-free medium in 24-well Matrigel-coated Transwell units with polycarbonate filters (CoStar Group Inc., Washington, DC, USA) containing 8-μm pores. The outer chambers were filled with 0.5 ml medium containing 10% FBS. At 24 h, the cells were fixed in methanol and stained with crystal violet. The top surface of the membrane was gently scrubbed with a cotton bud and cells invading through the membrane filters were counted on glass slides. Images were captured using an inverted microscope equipped with a CCD camera (IX71; Olympus Corp.).

### Xenograft tumour-growth assay

The protocol was approved by the ethics review committee for animal experimentation (Nantong University). In total, 16 BALB/C nude mice (SPF; 6 weeks-old; body weight, 20±3 g; Shanghai Super-B&K Laboratory Animal Co., Ltd., Shanghai, China) were randomly divided into mock, negative, shRNA and control groups (4 mice in each group). Cells (2×10^7^/mouse) suspended in 0.2 ml DMEM were injected subcutaneously into the right flank of the nude mice. Tumour size was measured using calipers at the indicated time points and their volume was calculated according to the formula: Volume = (length × width^2^)/2. Mice were sacrificed on day 21 following injection. The following formula was used to calculate the rate of tumourigenicity inhibition: Tumourigenicity inhibition rate = [(tumour weight control - tumour weight shRNA)/tumour weight control] × 100.

### Pathology and immunohistochemistry

Pathological examination was performed by H&E staining. Immunohistochemistry (streptavidin-peroxidase method) with anti-human ANXA2 antibody (1:500; Santa Cruz Biotechnology, Inc.) was performed and negative controls were treated with non-specific mouse IgG. ANXA2 staining was assessed using the immunoreactive score. In brief, the percentage of positive cells was semi-quantitatively classified as follows: Diffuse positive (+++), >50% of total cells; moderate (++), 16–50%; and weak (+), 5–15%; and negative (−), <5%. Cells were evaluated by two independent pathologists and any differences in interpretation were resolved by consensus. Duplicate tissue cores for each tumour showed high levels of homogeneity for staining intensity and percentage of positive cells. The higher score was used as the final score in cases with a difference between duplicate tissue cores.

### Western blotting

Tissues were homogenised in an ice-cold homogenisation buffer and centrifuged at 800 × g. Total proteins were determined by bicinchoninic acid assay. Each 20 mg of protein was separated by 15% SDS-PAGE and then transferred onto polyvinylidene fluoride membranes and blocked with 5% BSA in Tris-buffer. Membranes were immunoblotted overnight with anti-ANXA2 or anti-β-actin antibodies (Santa Cruz Biotechnology, Inc.), followed by respective horseradish peroxidase-conjugated secondary antibodies. Bands were subsequently visualised by a chemiluminescence detection system (Millipore, Billerica, MA, USA) and density was determined by an image analyser. ANXA2 levels were presented as relative ratio (RR) and calculated using the formula for signal intensity (SI) of ANXA2 and β-actin: RR = SI_ANXA2_/SI_β-actin_.

### Statistical analysis

MHCC97-H cells and xenograft tumours in nude mice were divided into shRNA, negative and mock groups. Statistical evaluation of results was performed by the least significant difference or Newman-Keuls test, Student’s t-test, χ^2^ test and Fisher’s exact test. P<0.05 was considered to indicate a statistically significant difference.

## Results

### Abnormal expression of ANXA2 in HCC patients

The intensity of ANXA2 expression in HCC, adjacent and distant cancerous specimens are shown in Table I. ANXA2 expression was markedly higher in HCC compared with that of the adjacent or distant cancerous tissues. ANXA2 was localised to the cell membrane and cytoplasm of HCC tissue (100%) and mainly in the cytoplasm of the matched adjacent tissue (90%), however, the distant cancerous tissue was not ANXA2-positive. Although no significant difference in the rate of ANXA2 expression (χ^2^=3.518; P=0.070) was found between the HCC and adjacent groups, the intensity of ANXA2 expression in the HCC group was significantly higher compared with that of the adjacent (Z=6.113; P<0.001) or distant cancerous groups (Z=7.328; P<0.001). A significant difference (F=498.221; P<0.001) was found among the various groups and ratio of ANXA2.

### Intervention of ANXA2 activation in hepatoma cells

**Figure 1:**
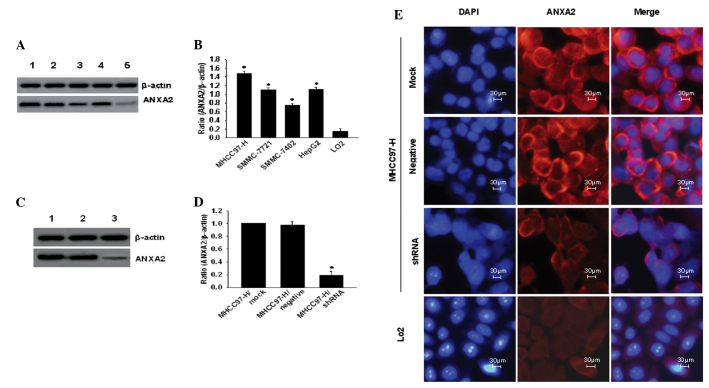
Expression of ANXA2 in hepatoma cell lines and downregulation of MHCC97-H cells following shRNA transfection. (A) ANXA2 expression in 5 cell lines: Lane 1, MHCC97-H; 2, SMMC-7721; 3, SMMC-7402; 4, HepG2; and 5, LO_2_. (B) Ratio of ANXA2 to β-actin in hepatoma cell lines (n=3). (C) ANXA2 expression in MHCC97-H cells following shRNA transfection. Lane 1, mock; 2, negative; and 3, shRNA. (D) Ratio of ANXA2 to β-actin (n=3). (E) Immunofluorescence analysis of ANXA2 expression in cells following shRNA transfection (magnification, ×400). ANXA2, Annexin A2; shRNA, short hairpin RNA.

Alterations in the expression of ANXA2 in the hepatoma cell lines with low or high metastatic potential following transfection with shRNA are shown in Fig. 1 and Table II. ANXA2 expression was significantly higher (P<0.05) in hepatoma cells with a 5–8 fold increased expression compared with that of the LO_2_ cells (Figs. 1A and 1B). ANXA2-mRNA levels in MHCC97-H cells were significantly higher (F=286.254; P<0.001) compared with that of the other cell lines (Table II). The MHCC97-H cells with the highest ANXA2-mRNA levels were selected to observe the effects of shRNA targeting ANXA2 on the proliferation and invasion of cells. ANXA2-mRNA was markedly downregulated (q=71.993; P<0.001) following transfection with stably expressing shRNA at the protein level (Fig. 1C). The ratio of ANXA2 to β-actin (Fig. 1D) demonstrated that ANXA2 expression in the shRNA group was significantly downregulated (P<0.001), with an evidently weaker red immunofluorescence signal (Fig. 1E) in the membrane and cytoplasm. Decreasing immunofluorescence was not noted in the negative or mock groups. However, the mock cells marked with DAPI dye showed a number of fragmented nuclei with condensed chromatin.

### Suppression of tumour cell proliferation

**Figure 2:**
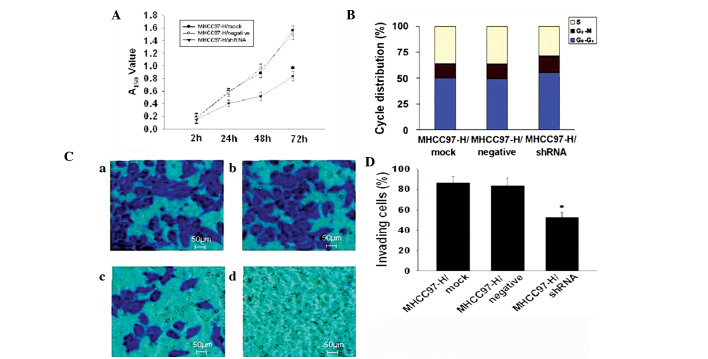
Effects of targeting ANXA2 gene transcription on MHCC97-H cell proliferation and cell cycle. (A) Proliferation assay using a CCK-8 at A450 (n=5). (B) Analysis of cell cycles by flow cytometry (n=3). (C) Alteration of MHCC97-H/shRNA cell invasive potential following shRNA transfection (magnification, ×400): (a) mock, (b) negative, (c) shRNA and (d) blank groups. (D) Decrease in cell invasive potential following shRNA transfection (n=3). ANXA2, Annexin A2; CCK-8, cell counting kit-8; A450; absorbance at 450nm; shRNA, short hairpin RNA.

The effect of ANXA2 suppression on the proliferation and cell cycle of MHCC97-H cells following transfection with specific shRNA is shown in Fig. 2. The cell proliferation ability (Fig. 2A) in the shRNA group was significantly decreased (P<0.05) compared with that of the mock and negative groups, but no significant difference was found between the mock and negative groups. Analysis of the cell cycle (Fig. 2B) indicated that the ratio of cells in S phase in the shRNA group was significantly decreased compared with the mock group [27.76 and 36.14%, respectively (P<0.05)] compared with that of the mock group, whereas the ratio of cells in G_0_–G_1_ and G_2_-M phases were increased compared with that of the mock group (P<0.05). The growth of shRNA cells was markedly inhibited *in vitro* and the MHCC97-H cell invasive potential was significantly downregulated (Fig. 2D; P<0.05) in the shRNA group compared with that of the mock cells (percentage of invading cells, 52.16 and 86.14%, respectively; Fig. 2D).

### Silencing ANXA2 expression in xenograft tumours

**Figure 3:**
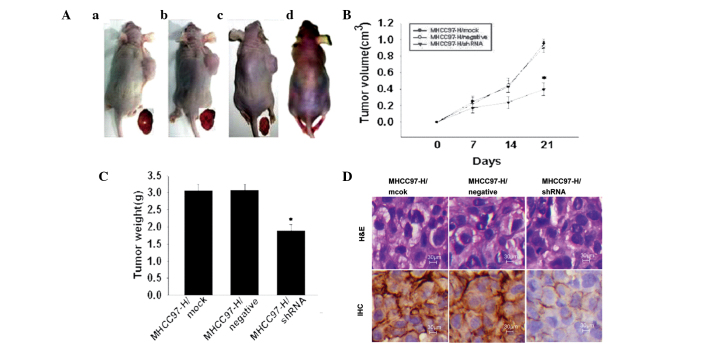
Silencing ANXA2 gene transcription in the subcutaneous xenograft tumours with MHCC97-H/shRNA cell tumourigenicity *in vivo*. (A) Tumourigenic nude mice and tumours and controls. The mice appeared to be evidently emaciated. (a) MHCC97-H/mock mouse; (b) MHCC97-H/negative mouse; (c) MHCC97-H/shRNA mouse; and (d) control mouse. (B) Tumour growth of the shRNA group and (C) alteration of tumour weights in the various groups. (D) Analysis of ANXA2 expression by immunohistochemistry in the xenograft tumours (magnification, ×400). ANXA2, Annexin A2; shRNA, short hairpin RNA.

The effect of silencing ANXA2 in xenograft tumours on tumour growth *in vivo* is shown in Fig. 3. Tumourigenicity (Fig. 3A) showed a marked reduction in tumour size in the shRNA group compared with that of the mock or negative groups and the rate of tumour inhibition was 38.24%. The tumour growth curve (Fig. 3B) over 21 days indicates that silencing ANXA2 reduced the tumourigenic potential (P<0.05) *in vivo.* The mice appeared emaciated and tumour weight was 1.89±0.16 g in the shRNA group (Fig. 3C.), significantly lower compared with that of the mock (3.06±0.14 g) and negative (3.07±0.17 g) groups. Pathological analysis of the tumours showed no clear morphological alterations (H&E; Fig. 3D) and the distribution of ANXA2 was mainly localised to the cell membrane in the shRNA group and the cell membrane and cytoplasm in the mock and negative groups. ANXA2 intensity in tumours was significantly lower (Z=2.530; P=0.011) in the shRNA group (3/4; +) compared with that of the mock (4/4; ++ and +++) and negative (4/4; +++) groups.

## Discussion

HCC is characterised by a multi-cause, multi-stage and multi-focus process of tumour progression (1,4) and poor prognosis. Therefore, early diagnosis and therapy of HCC are of utmost importance (6). The diagnostic values of ANXA2 have been previously reported as useful potential markers for HCC (23). In the present study, ANXA2 was overexpressed in HCC tissues and although no significant difference in ANXA2 expression was found between the HCC and adjacent cancerous groups, its intensity was significantly higher compared with that of the adjacent or distant cancerous groups. ANXA2 expression in the adjacent tissue presented in an intermediate state and may be the result of the microenvironment and cell transformation. This state promotes metastasis and leads to the activation of MMPs and degradation of ECM components.

ANXA2 is upregulated in HBV- and/or HCV-associated HCC. ANXA2 induces cell migration and neoangiogenesis via tissue plasminogen activator-dependent plasmin generation and represents the metastatic potential of HCC (14,24). ANXA2 levels have been previously investigated in HCC and benign liver diseases. The incidence of ANXA2 abnormality was 86.96% in HCC and 80% in metastatic liver cancer and significantly higher compared with that of benign liver diseases or controls. The clinicopathological features of ANXA2 demonstrated that there is a close correlation between ANXA2 expression in patients with HCC and metastasis. ANXA2 has been correlated with HBV, extrahepatic metastasis, portal vein thrombus, differentiated grading and TNM staging. However, no significant correlation has been found between ANXA2 and tumour size or AFP. In addition, no statistically significant difference has been found between moderately- and poorly-differentiated HCC or TNM stages III and IV, which indicates that abnormal ANXA2 may be associated with a poor prognosis in HCC (21).

Metastasis remains a major challenge in the management of HCC. Accumulating evidence indicates that interactions between ANXA2 and its binding proteins are important for the tumour microenvironment and function together to enhance metastasis (17). The highest levels of ANXA2 expression were confirmed in MHCC97-H cells with high metastatic potential and ANXA2 expression is associated with high metastatic potential and invasion ability of hepatoma cells. Application of shRNA may effectively target ANXA2 in MHCC97-H cells *in vitro*. Cell proliferation ability was significantly decreased in the shRNA group with a lower ratio of cells in the S phase, whereas the ratio of cells in the G_0_–G_1_ and G_2_-M phases were found to increase. Apoptosis occurrence was observed in shRNA and LO_2_ cells and the proliferation of high metastatic potential cells was markedly suppressed by the apoptosis mechanism, with significantly decreased invasive ability and growth inhibition (Fig. 2). The ANXA2 gene may be a potential therapeutic target for HCC metastasis.

ANXA2 is a novel signalling mediator of HBV-related chronic inflammation-induced tumour metastasis (25). In the present study, the tumourigenic nude mice appeared to be markedly emaciated *in vivo*, particularly in the mock and negative groups, but not in the shRNA group (Fig. 3). Tumour weights of the shRNA group showed a significant decrease compared with that of the mock group, with measuring the tumour volume at various times and tissue expression of ANXA2. ANXA2 distribution was mainly localised in the cell membrane and cytoplasm, but negligible in the cell nucleus. No statistically significant difference was found between the mock and negative groups, indicating that shRNA may downregulate tumour ANXA2 expression and suppress tumour growth *in vivo*.

In conclusion, in the present study, abnormal ANXA2 expression was found to correlate with HBV, extrahepatic metastasis and portal vein thrombus. ANXA2 was upregulated in cells with high metastatic potential. In the snRNA group, inhibition of ANXA2 was found to arrest the cell cycle *in vitro* and inhibit tumour growth *in vivo*, which highlighted further insight into understanding the biological features of HCC with high metastatic potential. The results not only revealed an association between ANXA2 and HCC metastasis but also highlighted a potential therapeutic target for HCC. Therefore, it is important that further investigations are performed to identify additional signalling modulators and delineate the pivotal regulatory mechanisms involving ANXA2 and metastasis (26–28).

## Figures and Tables

**Table I:** Expression of ANXA2 in HCC, adjacent and distant cancerous tissues.

		ANXA2 intensity					
				
Cancerous tissue group	n	−, n	+, n	++, n	+++, n	Z	P-value
HCC	30	0	1	7	22		
Adjacent	30	3	16	11	0	6.113^a^	<0.001
Distant	30	30	0	0	0	7.328^a^	<0.001

a Compared with the HCC tissue group.

ANXA2, Annexin A2; HCC, hepatocellular carcinoma; −, negative; +, weak; ++, moderate, +++, positive.

**Table II:** ANXA2 mRNA expression in HCC lines and inhibition of ANXA2 gene transcription in MHCC97-H cells with shRNA transfection.

Group	n	Ct_ANXA2_	Ct_β-actin_	2^−ΔΔCt^	q	P-value
HCC lines						
LO_2_	5	25.16±0.09	20.86±0.03	1.00		
HepG2	5	22.14±0.15	20.66±0.02	7.07±0.35	32.200^a^	<0.001
SMMC-7402	5	22.87±0.15	20.80±0.14	4.68±0.31	19.517^a^	<0.001
SMMC-7721	5	22.21±0.12	20.72±0.10	7.02±0.19	31.923^a^	<0.001
MHCC97-H	5	21.85±0.26	20.78±0.13	9.45±0.53	44.814^a^	<0.001
shRNA transfection						
MHCC97-H/mock	5	21.84±0.11	20.77±0.16	1.00		
MHCC97-H/negative	5	21.83±0.21	20.72±0.10	0.97±0.04	2.922^b^	0.073
MHCC97-H/shRNA	5	24.24±0.55	20.80±0.14	0.20±0.05	71.793^b^	<0.001

Compared with the ^a^LO_2_ and ^b^MHCC97-H/mock groups. ANXA2, Annexin A2; HCC, hepatocellular carcinoma; shRNA, short hairpin RNA.
